# Capturing change in restricted and repetitive behaviour in preschoolers with ASD: A comparison of direct behavioural observation and parent report

**DOI:** 10.1111/jcpp.70009

**Published:** 2025-07-09

**Authors:** Naisan Raji, Janina Kitzerow‐Cleven, Ziyon Kim, Solvejg K. Kleber, Leonie Polzer, Christian Lemler, Melanie Ring, Regina Taurines, Julia Geißler, Ulrike Fröhlich, Michele Noterdaeme, Nico Bast, Christine M. Freitag

**Affiliations:** ^1^ Department of Child and Adolescent Psychiatry, Psychosomatics and Psychotherapy University Hospital Frankfurt Frankfurt Germany; ^2^ Department of Child and Adolescent Psychiatry, Psychosomatics and Psychotherapy University Hospital Wuerzburg Wuerzburg Germany; ^3^ Department of Child and Adolescent Psychiatry and Psychotherapy University Hospital Dresden Dresden Germany; ^4^ Department of Child and Adolescent Psychiatry and Psychotherapy Josefinum Augsburg Augsburg Germany

**Keywords:** Autism spectrum disorders, longitudinal studies, development, behavioural measures, stereotyped behaviour

## Abstract

**Background:**

Restricted and repetitive behaviour (RRB) in autism spectrum disorder (ASD) can be assessed by different measures, which diverge in item quantity, dimensionality or source of information. However, change sensitivity has not been systematically investigated among commonly used measures, albeit its importance for clinical trials and longitudinal studies.

**Methods:**

Longitudinal data resulting from behavioural observation (Autism Diagnostic Observation Schedule‐2, ADOS‐2; Brief Observation of Social Communication Change, BOSCC) and parent report (Restricted Behaviour Scale‐Revised, RBS‐R) was collected for 134 toddlers and preschoolers aged 25–65 months diagnosed with ASD by the Autism Diagnostic Interview‐Revised (ADI‐R) and ADOS‐2. Change sensitivity was estimated using the reliable‐change index and developmental trajectories of RRB by linear mixed models and k‐means clustering.

**Results:**

The RBS‐R identified significantly more reliable change in RRB severity compared to ADOS‐2 and BOSCC. For all measures, except the RBS‐R self‐injurious behaviour subscale, three distinct RRB trajectories were found as follows: increasing, stable and decreasing RRB severity. Overlap was low between trajectory group assignment across measures, as were cross‐sectional correlations between ADI‐R, ADOS‐2, BOSCC and RBS‐R. Trajectory group comparisons among measures mostly showed lower baseline RRB severity in the increasing trajectory groups and higher baseline RRB severity in the decreasing trajectory groups. The trajectory groups did not differ in age or nonverbal IQ across RRB measures, except for the RBS‐R compulsive behaviour subscale, which had higher nonverbal IQ in the decreasing trajectory group.

**Conclusions:**

The dimensional questionnaire RBS‐R compared to ADOS‐2 and BOSCC is superior in capturing subtle changes in RRB during preschool age.

## Introduction

Restricted and repetitive behaviours (RRB) encompass a broad variety of behaviours, such as repetitive motor movements, special interests or insistence on sameness. RRB are observed in neurotypically developing children (Larkin, Meins, & Leekam, 2019), but pervasively occur in children with intellectual disability, obsessive–compulsive disorder or anxiety (Baribeau et al., 2021; Jiujias, Kelley, & Hall, 2017; Kästel et al., 2021).

RRB are also a core symptom domain of Autism Spectrum Disorder (ASD), alongside social communication (American Psychiatric Association, 2013). According to DSM‐5, ASD‐related RRB are: (1) stereotyped or repetitive motor movements or use of objects and speech, (2) insistence on sameness behaviours such as adherence to routines and ritualised behaviours, (3) restricted interests and (4) atypical sensory responses such as hyper‐ or hyporeactivity or sensory interests.

Measurement of RRB is crucial for understanding the severity and impact of these behaviours and for monitoring treatment effect. Although a large body of literature on RRB in ASD has been published since the implementation of the DSM‐5 (Iversen & Lewis, 2021), there is no gold‐standard RRB assessment. RRB measures vary widely in item quantity, item content and source of information, which in turn affect psychometric properties and, potentially, change sensitivity. The latter is especially crucial for the evaluation of interventions that require outcomes sensitive enough to capture change within shorter time spans. In this study, we aim to explore 1‐year change sensitivity of different RRB measures in individuals with ASD.

A commonly reported RRB measure is the Autism Diagnostic Observation Schedule (current version: ADOS‐2; Lord et al., 2012), which has been developed as a diagnostic instrument for ASD. The ADOS‐2 is a direct behavioural observation measure based on semistructured play and dialogues. Varying by language‐level module, its RRB subscale contains 4 to 5 items describing behaviours such as repetitive interests and stereotyped motor movements, hand and finger or body mannerisms, intonation of vocalisations or verbalisations, stereotyped speech or sensory interests. Factor analyses applied to the ADOS(‐2) RRB items have not converged since each of the items pools a variety of behaviours (Uljarević et al., 2021). Across modules, a standardised ADOS‐2 RRB score has been developed to provide a measure less influenced by child characteristics (Calibrated Severity Score, CSS; Hus, Gotham, & Lord, 2014). Based on the ADOS‐2, the Brief Observation of Social Communication Change (BOSCC; Grzadzinski et al., 2016) was developed as a behavioural observation to detect change in core ASD symptoms over time. Its RRB subscale assesses repetitive interests and stereotyped behaviour, hand and finger or body movements as well as sensory interests and repetitive play with objects. The 4 items are rated using a decision tree that captures quality and frequency of certain behaviours. Several behaviours are rated together to score a specific item, while a factor structure remains to be investigated.

In contrast to observation‐based measures administered and coded by trained clinicians, questionnaires such as the Repetitive Behaviour Scale‐Revised (Lam & Aman, 2007) are cost‐effective tools suited to extend the scope of RRB research to trans‐diagnostic samples (Boyd et al., 2010; Kästel et al., 2021). The RBS‐R contains 43 items and captures a broad spectrum of RRB by caregiver, (kindergarten‐) teacher or self‐report. The dimensions of the RBS‐R have often been studied (Hooker, Dow, Morgan, Schatschneider, & Wetherby, 2019; Kästel et al., 2021; Lam & Aman, 2007; Sturm, Huang, & Kuhfeld, 2022). In the largest study with mixed clinical and control samples, four RRB‐related factors were described with persistent (synonymous to IS), stereotyped behaviour (synonymous to RSM), self‐injurious behaviour (SI) and compulsive behaviour (CB; Kästel et al., 2021).

Previous studies have shown weak to moderate cross‐sectional correlations between ADOS‐2‐RRB (toddler module) and RBS‐R RSM (*r* = .19, Schertz, Odom, Baggett, & Sideris, 2016) and ADOS‐RRB and BOSCC‐RRB (*r* = .36, Pijl et al., 2018), suggesting that each measure may capture different aspects of RRB in individuals with ASD.

Longitudinally, ADOS(‐2), BOSCC and RBS‐R demonstrated some degree of change sensitivity. The ADOS(‐2) has been shown to capture RRB change in an intervention study in preschoolers (Green et al., 2010). Longitudinal studies have been describing trajectories of RRB development from preschool to school age, with decreasing and stable RRB trajectories (Franchini et al., 2023) or decreasing, increasing and stable trajectories (Waizbard‐Bartov et al., 2022). The BOSCC demonstrated RRB change in studies with small sample sizes (Kim, Grzadzinski, Martinez, & Lord, 2019; Pijl et al., 2018), but not in a large study with preschoolers (Carruthers et al., 2021). The RBS‐R has been widely employed to explore the dimensionality of the RRB construct (McDermott, Farmer, Gotham, & Bal, 2020) and has proven useful in identifying distinct longitudinal RRB severity profiles during infancy (Wolff et al., 2014). It has not yet been included as an outcome measure in large‐scale early intervention studies.

Taken together, previous literature shows that the ADOS‐2, BOSCC and RBS‐R may be useful as change‐sensitive outcome measures of RRB; still, study results were constrained by study design, sample size and age range of participants. This study aims to compare RRB change sensitivity of these measures in toddlers and preschoolers. Given the importance of change sensitivity for intervention studies, this research is especially relevant for future clinical trial designs. Based on the reported literature, we expect first a difference in change sensitivity between ADOS‐2, BOSCC and RBS‐R and second heterogeneity of individual trajectories based on the three measures.

## Methods

### Participants

Participants were recruited from four German (University) Hospital Departments of Child and Adolescent Psychiatry, Psychosomatics and Psychotherapy within the A‐FFIP early intervention trial (Kitzerow et al., 2020). A total of 134 children aged between 25 and 65 months with ASD according to DSM‐5 were included (see Table 1). Since we were not interested in intervention effects, we pooled participants from the randomised controlled trial. Diagnoses were confirmed by trained psychologists or psychiatrists based on a standardised procedure using the German versions of the ADOS‐2 (Poustka et al., 2015) and the Autism Diagnostic Interview‐Revised (ADI‐R; Bölte, Poustka, Rühl, & Schmötzer, 2006) or the ADI‐R toddler algorithm in children younger than 4 years (Kim & Lord, 2012). ADI‐R, ADOS‐2, BOSCC, RBS‐R and cognitive performance were assessed at baseline (T1). Additional assessments of ADOS‐2, BOSCC and RBS‐R were conducted approximately 7 (T4) and 14 months (T6) after baseline. For exact periods between timepoints, see Table S1.

**Table 1:** Sample description (baseline)

	*n* (%)	*M*	*SD*	Range
Age (months)	134	48.9	10.2	25–65
Sex (female)	26 (19)			
Migration status (yes)	80 (63)			
ISCED	134	2.7	1.0	0–5
Verbal IQ	134	48.9	27.0	16–121
Nonverbal IQ	134	61.3	20.8	31–123
ADI‐R Toddler (age < 4) total	64 (48)			
12–20 months/nonverbal 21–47 months	29	18.9	3.1	11–25
Some words 21–47 months	19	17.9	4.5	11–25
Phrase speech 21–47 months	16	19.9	5.4	13–30
ADI‐R (age ≥ 4) RRB domain	70 (52)	5.6	2.2	2–11
ADOS‐2 CSS total	134	7.3	1.6	4–10
ADOS‐2 module 1	103 (77)			
ADOS‐2 module 2	24 (18)			
ADOS‐2 module 3	11 (8)			

Migration status was considered positive if at least one parent had immigrated to Germany as an adult. No information on migration status was available in 6 cases. ISCED, International Standard Classification of Education (Index on parental education (OECD, http://www.oecd.org/education/1841854.pdf)). ADI‐R, Autism Diagnostic Interview‐Revised; ADOS‐2, Autism Diagnostic Observations Schedule‐2; CSS, calibrated severity score.

### Measures

#### ADOS‐2

The ADOS‐2 is a standardised behaviour observation measure to score ASD symptom domains social communication and RRB based on clinician‐child interaction during standardised activities. We used modules for nonverbal to minimally verbal children (M1), phrase speech (M2) and fluent speech (M3). Within 1 year, 10 children switched from M1 to M2, and 4 children switched from M2 to M3. Algorithm items were video‐coded by trained staff, and the CSS‐RRB was derived across modules (Hus et al., 2014). Internal consistencies were low for M1 (*α* = .21) and M2 (*α* = .19) and acceptable for M3 (*α* = .54). Double coding was done for 10% of all assessments. Inter‐rater reliability was established by intraclass correlation (ICC; see Appendix S1) and was acceptable (ICC = .47, 95% CI 0.20, 0.67).

#### BOSCC

The BOSCC (December 2017 version) is a standardised behaviour observation scale scoring items in social communication and RRB based on adult–child interaction during free play with standardised sets of toys. Videotaped interactions with trained staff unknown to the child (two 6‐min segments) were coded by independent researchers on four RRB items on a scale ranging from 0 to 5. Item scores per 6‐min segment were averaged and aggregated into one RRB domain score ranging from 0 to 20. The BOSCC was administered and coded under supervision by a coauthor (JKC) trained by the BOSCC authors. Double coding for 10% of all assessments showed good inter‐rater reliability (ICC = .89, 95% CI 0.88, 0.90). Internal consistency of the RRB subscale was *α* = .49 in this study. The BOSCC version used in this study was designed and validated for minimally verbal children (Grzadzinski et al., 2016). Our study also included children with phrase and fluent speech (number of BOSCC: *n*_T1_ = 30 [23%], *n*_T4_ = 34 [28%], *n*_T6_ = 41 [35%]), operationalised by assessment with the ADOS‐2 modules M2 and M3. Hence, we additionally conducted all analyses related to the BOSCC for the subset of minimally verbal children (ADOS‐2 module M1) at the respective timepoints (number of BOSCC: *n*_T1_ = 99 [77%], *n*_T4_ = 86 [72%], *n*_T6_ = 77 [65%]).

#### RBS‐R

The RBS‐R is a rating scale with 43 items coded on a scale from 0 to 3. In this study, the RBS‐R was completed by the primary caregiver. Additionally, to a total score, we studied four subscales (IS, RSM, SI, CB; Kästel et al., 2021). Internal consistency of the RBS‐R in this study was excellent (total score: *α* = .94; IS: *α* = .90) to good (RSM: *α* = .81; SI: *α* = .80; CB: *α* = .83).

#### Additional measures

As an additional baseline RRB measure, the ADI‐R‐RRB was assessed. It comprises eight items related to the four DSM‐IV‐TR RRB areas: circumscribed interests, adherence to routines, stereotyped motor movements and preoccupation with parts of objects, rated by severity on a scale from 0 to 2. We used the current scores of children aged 4 years and above, since the toddler algorithms do not provide an RRB score.

The educational status of the parents was classified using the International Standard Classification of Education (ISCED, http://www.oecd.org/education/1841854.pdf). Verbal (VIQ) and nonverbal IQ (NVIQ) were assessed by the German versions of the Bayley‐III (Reuner & Rosenkranz, 2014) or the WPPSI‐III (Petermann, Ricken, Fritz, Schuck, & Preuss, 2014), depending on developmental age (Kitzerow et al., 2020).

### Data analysis

All statistical analyses were performed using R‐4.2.1 (R Core Team, 2022) with the additional packages lme4 (Bates, Mächler, Bolker, & Walker, 2015), lmerTest (Kuznetsova, Brockhoff, & Christensen, 2017), ggplot2 (Wickham, 2016), ggpubr (Kassambara, 2023) and a customised function based on JTRCI (Kruijt, 2022). The research plan was preregistered prior to accessing the data (Raji et al., 2023; https://osf.io/fx2yq). Spearman rank correlations were used to describe the associations between ADI‐R‐RRB, ADOS‐2‐CSS‐RRB, BOSCC‐RRB and RBS‐R at baseline. Change sensitivity across the longitudinally assessed measurements was studied by two complementary approaches. First, we calculated the reliable‐change index (RCI; Jacobson & Truax, 1991) using the absolute pairwise change scores between timepoints for each instrument (for detailed description see Appendix S2). Based on the RCI, participants were allocated to the groups reliable symptom decrease, reliable symptom increase and no reliable change (i.e. the change did not exceed the error rate, *α* = .05). FDR‐corrected pairwise Chi^2^ tests were performed to test for differences between the instruments regarding the three RCI‐based groups. Second, we applied linear mixed models (LMM) to investigate repeated assessments across participants. We specified one LMM per measure (ADOS‐2‐CSS‐RRB, BOSCC‐RRB, RBS‐R total score, RBS‐R subscales). Timepoint was the predictor of interest and was specified as a linear effect after visual inspection. Sex, age, ISCED, NVIQ and study site were included as fixed‐effect covariates. To allow for better interpretation of intercepts, sex and age were grand‐mean‐centred. All models included random intercepts for participant and random slopes for participant over time to account for subject‐specific RRB trajectories. Significant differences in fixed effects of time between measures were determined by nonoverlapping of the 95% confidence intervals (CI).

Heterogeneity of individual trajectories was explored using k‐means clustering based on the standardised slopes derived from the respective LMM (Grzadzinski et al., 2023). The k‐means algorithm was run using different values for *k* (1–8), each run using 10 random sets of initial cluster centres. We determined the adequate number of clusters by comparing BICs and by evaluation of cluster sizes and interpretability of the clusters.

## Results

Baseline correlations between the measures are shown in Table 2. ADI‐R‐RRB was moderately correlated with ADOS‐2‐CSS‐RRB and RBS‐R scores except SI. ADOS‐2‐CSS‐RRB did not correlate with BOSCC‐RRB or RBS‐R. BOSCC‐RRB negatively correlated with RBS‐R scores and showed no correlation with ADI‐R‐RRB. In the subsample of minimally verbal children, the BOSCC‐RRB showed a weak positive correlation (*ρ* = .24, 95% CI 0.02, 0.42) with the ADOS‐2‐CSS‐RRB, but not with the ADI‐R or the RBS‐R.

**Table 2:** Spearman rank correlations between RRB subscales at baseline (T1)

	ADI‐R	ADOS‐2‐RRB‐CSS	BOSCC‐RRB	RBS‐R	RBS‐R IS	RBS‐R	RBS‐R SI
						RSM	
ADOS‐2‐RRB‐CSS	.40**						
BOSCC‐RRB	.01	.16					
RBS‐R	.49***	.01	−.21*				
RBS‐R IS	.40**	−.04	−.33***	.91***			
RBS‐R RSM	.33*	.11	.14	.73***	.45***		
RBS‐R SI	.21	.09	−.11	.48***	.33***	.31**	
RSBR CB	.47***	0	−.22*	.85***	.72***	.54***	.40****

ADI‐R, Autism Diagnostic Interview‐Revised; ADOS‐2, Autism Diagnostic Observations Schedule‐2; CSS, calibrated severity score; BOSCC, Brief Observation of Social Communication Change; RBS‐R, Repetitive Behaviour Scale‐Revised; IS, insistence on sameness; RSM, repetitive sensorimotor movements; SI, self‐injurious behaviour; CB, compulsive behaviour. Correlations with the ADI‐R‐RRB based on children ≥4 years of age (*n* = 70). *p < .05, **p < .01, ***p < .001. All *p* values were FDR‐adjusted.

### Sensitivity to detect change

**Figure 1:**
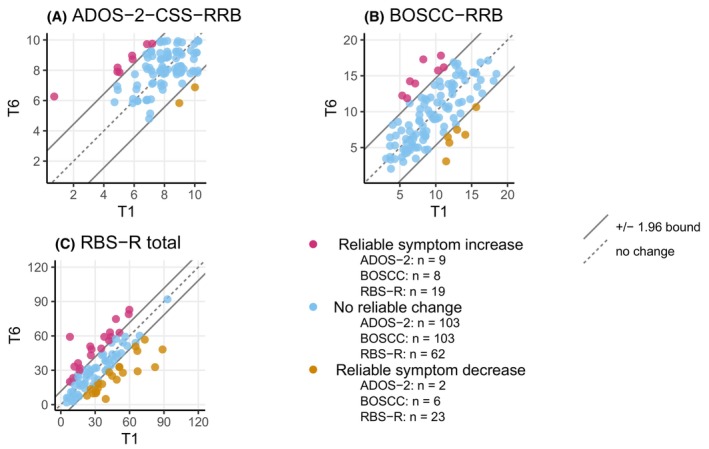
Change groups based on the reliable‐change index (T1–T6) by instrument *x*‐Axis depicts baseline scores (T1), *y*‐axis depicts scores at T6. ADOS‐2, Autism Diagnostic Observation Schedule‐2; BOSCC, Brief Observation of Social Communication Change; CSS, calibrated severity score; RBS‐R, Repetitive Behaviour Scale‐Revised. For better visual representation of the complete sample, dots were allowed to jitter around the actual values to prevent overlap. The 1.96 error band represents *α* = .05

Mean and *SD* of all measures between timepoints are presented in Table S2. Figure 1 shows the scores of the ADOS‐2‐CSS‐RRB, BOSCC‐RRB and RBS‐R total score and the corresponding RCI‐based reliable‐change groups (reliable symptom increase, no reliable change, reliable symptom decrease) at timepoints T1 and T6. Post hoc Chi^2^ tests indicated the superior ability of the RBS‐R to capture change in contrast to ADOS‐2 and BOSCC (full sample). Comparing ADOS‐2‐CSS‐RRB and RBS‐R, differences regarding the three reliable‐change groups were significant for the RBS‐R total score (*χ* ^2^(2) = 31.01, *p* < .01) and the subscales IS (*χ* ^2^(2) = 17.62, *p* < .01), RSM (*χ* ^2^(2) = 13.29, *p* < .01) and CB (*χ* ^2^(2) = 8.46, *p =* .01), but not the RBS‐R SI subscale. Comparing BOSCC‐RRB and RBS‐R, significant differences were found regarding the RBS‐R total (*χ* ^2^(2) = 23.95, *p* < .01), subscales IS (*χ* ^2^(2) = 11.29, *p* < .01) and RSM (*χ* ^2^(2) = 6.35, *p =* .04). ADOS‐2‐CSS‐RRB and BOSCC‐RRB did not significantly differ regarding the distribution of the three categories (*χ* ^2^(2) = 2.02, *p =* .36). The absolute counts and percentages of reliable symptom increase, decrease or no reliable change over all time periods and in all subscales can be found in Table S3. Overlap of individuals categorised into the three reliable‐change groups between pairwise instruments is shown in Tables S4–S6. Overall, most overlap between instruments occurred in the no‐reliable‐change group. Only two participants showed a reliable symptom increase by one measure and a reliable symptom decrease by another measure in pairwise comparisons.

**Figure 2:**
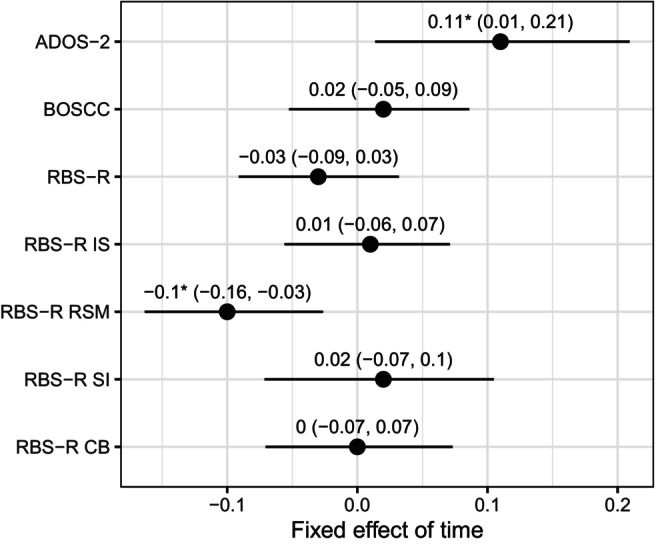
Time effects by RRB measure in linear mixed models ADOS‐2, Autism Diagnostic Observation Schedule‐2; BOSCC, Brief Observation of Social Communication Change; CB, compulsive behaviour; IS, insistence on sameness; RBS‐R, Repetitive Behaviour Scale‐Revised; RSM, repetitive sensorimotor movements; SI, self‐injurious behaviour. The error bars represent the 95%‐confidence intervals. *p < .05

Comparing absolute change over 14 months based on LMM, the ADOS‐2‐CSS‐RRB (*β* = .11, 95% CI 0.01, 0.21) and the RBS‐R RSM (*β* = −.10, 95% CI −0.16, −0.03) showed change sensitivity. For all other measures, the 95% CI of the time estimates included zero and did not significantly differ from each other (Figure 2).

The full model parameter estimates of the LMM are shown in the supporting information for ADOS‐2, BOSCC (full sample and minimally verbal subsample), RBS‐R total (Table S7) and the RBS‐R subscales (Table S8). Notably, younger children showed higher ADOS‐2‐RRB‐CSS and BOSCC‐RRB (full sample), and boys showed higher RBS‐R RSM scores.

### Cluster analysis of LMM‐based individual RRB trajectories

**Figure 3:**
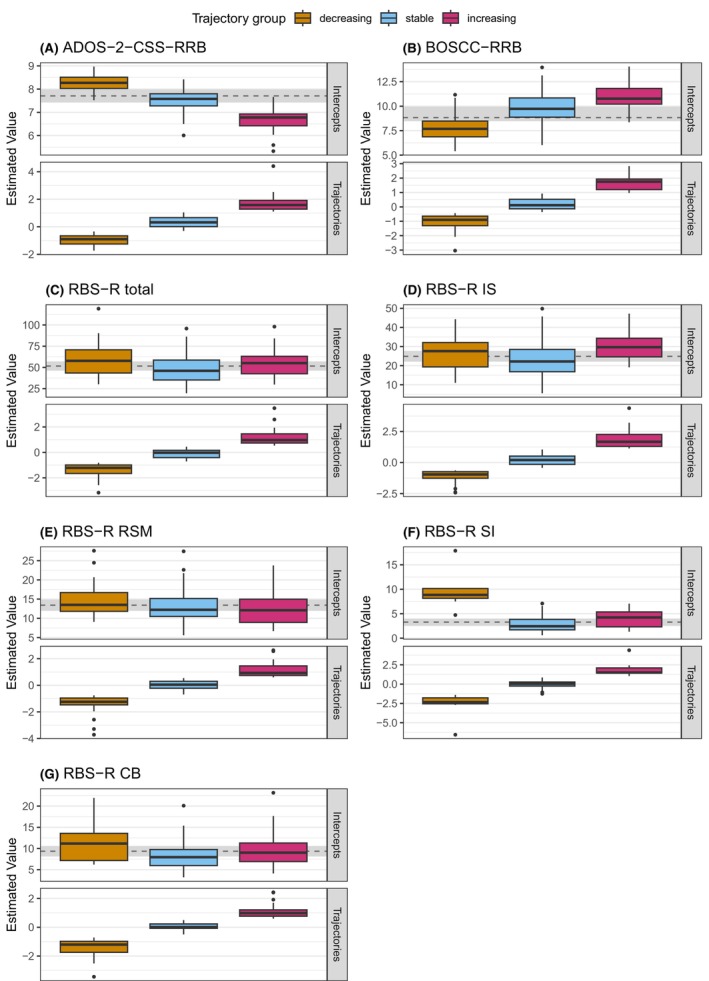
Intercepts and trajectories per trajectory group by instrument. The upper part depicts the linear mixed model‐based estimated intercept of symptom severity in the original metric of the instrument (full model parameters in Table S6). The lower part shows the standardised estimated trajectory. Outliers were not excluded since extreme symptom expression in RRB is clinically plausible, and the assignment to trajectory groups was checked and found correct. ADOS‐2, Autism Diagnostic Observation Schedule‐2; BOSCC, Brief Observation of Social Communication Change; CB, compulsive behaviour; CSS, calibrated severity score; IS, insistence on sameness; RBS‐R, Repetitive Behaviour Scale‐Revised; RSM, repetitive sensorimotor movements; SI, self‐injurious behaviour

Clustering the standardised LMM‐slopes, the BICs pointed to three to five cluster solutions based on visual inspection by inflexion points (Figure S1). Based on cluster sizes, we found three distinct individual‐trajectory groups as the best solution for all measures (Figure 3, Tables S9 and S10 with BIC estimates and cluster size ranges per cluster number).

Individual‐trajectory group 1 (‘decreasing’) was characterised by decreasing RRB symptoms over 14 months. Individual‐trajectory group 2 (‘stable’) showed stable RRB, and individual‐trajectory group 3 (‘increasing’) experienced increasing RRB symptoms. Using the ADOS‐2, 53 participants (40%) were assigned to the decreasing group, 65 participants (48%) remained stable, and 16 participants (12%) experienced an increase. In the BOSCC, 45 participants (34%) decreased, 67 participants (51%) remained stable, and 19 (15%) experienced increasing symptoms. Based on the RBS‐R total score, 24 participants (19%) showed decreasing RRB scores while 68 participants (53%) were assigned to the stable and 36 participants (28%) to the increasing group. The number of participants per trajectory group in the RBS‐R subscales are shown in Table S11. The RBS‐R SI subscale did not show a robust cluster solution, given that only 15 participants (11%) were in either the increasing or the decreasing trajectory group.

Overlap of individuals categorised into the three individual‐trajectory groups (decreasing, stable and increasing) between pairwise instruments is shown in Tables S12–S14. The highest overlap occurred in combinations of the stable trajectory group with any of the other individual‐trajectory groups (increasing, stable and decreasing). Conflicting trajectory group assignments (increasing in one and decreasing in the other instrument) occurred in a maximum of 13 participants in pairwise comparisons.

### Comparison of reliable‐change versus individual‐trajectory group assignments

Additionally, we compared the overlap of change group assignment across the two grouping methods (i.e. RCI‐based vs. clustering the LMM‐slopes) by instruments using Cohen's unweighted *κ*. We found the overlap to be low in the ADOS‐2‐CSS‐RRB (*κ* = .17, 95% CI 0.05, 0.28), fair in the BOSCC‐RRB (*κ* = .23, 95% CI 0.11, 0.35) and substantial in the RBS‐R (*κ* = .83, 95% CI 0.72, 0.92).

### Group characteristics of the change groups by instrument and group assignment method

To further describe the characteristics of the respective reliable‐change or individual‐trajectory groups, first, reliable‐change groups derived from the RCI‐analysis were compared regarding baseline scores, age and NVIQ using ANOVA. Results are shown in Table 3 and Table S15 (RBS‐R subscales). The reliable‐change groups showed significant differences in baseline scores across instruments. In general, lower scores were observed in the reliable‐increase groups and higher scores in the reliable‐decrease groups. Post hoc Tukey tests were conducted to determine which specific groups differed from each other (Table S16) and revealed that, using the ADOS‐2‐CSS‐RRB, the reliable‐increase group showed lower scores than the other groups while using the BOSCC‐RRB and RBS‐R, the reliable‐decrease group showed higher scores than the other groups. Next, comparing the LMM‐slope derived individual‐trajectory groups regarding baseline score, age and NVIQ, we also found significant differences in baseline scores between trajectory groups across instruments (Table 4; Table S11 for RBS‐R subscales). Similar to the group comparisons of the reliable‐change groups, the baseline scores were lower in the increasing individual‐trajectory group and higher in the decreasing individual‐trajectory group for ADOS‐2‐CSS‐RRB and RBS‐R total score and subscales. Based on Tukey tests, all ADOS‐2‐CSS‐RRB groups differed significantly from each other in the mentioned direction, while for the RBS‐R, only the decreasing individual‐trajectory group showed higher baseline scores (Table S17). For the BOSCC‐RRB individual‐trajectory groups, the opposite was observed, that is participants in the decreasing individual‐trajectory group had lower baseline scores than participants in the increasing and stable individual‐trajectory groups (see Figure 3b). This pattern was also observed in the minimally verbal subgroup.

**Table 3:** Baseline characteristics by instruments and reliable‐change group

	Reliable‐change group			ANOVA	
	Reliable symptom increase	No reliable change	Reliable symptom decrease	*F*	*p*
ADOS2CSSRRB	*n* = 9	*n* = 103	*n* = 2		
Baseline	5.4 (1.9)	8.2 (1.3)	9.5 (0.7)	19.44	<.001
Age (months)	50.2 (9.5)	49.7 (10.5)	38.5 (2.1)	1.17	.32
NVIQ	59.7 (20.5)	60.9 (20.7)	92 (7.1)	2.27	.11
BOSCC‐RRB	*n* = 8	*n* = 103	*n* = 6		
Baseline	8.19 (2.31)	9.46 (3.72)	12.90 (1.59)	3.27	.04
Age (months)	50.80 (8.68)	49.50 (10.70)	47.50 (9.42)	0.16	.85
NVIQ	56.9 (17.3)	61.6 (21)	55.9 (17.3)	0.45	.64
BOSCC‐RRB MV	*n* = 7	*n* = 77	*n* = 6		
Baseline	8.57 (2.21)	10.40 (3.51)	12.90 (1.59)	2.73	.07
Age (months)	50.9 (9.37)	48.90 (10.60)	47.5 (9.42)	0.34	.71
NVIQ	53.60 (15.80)	53.3 (14.7)	55 (21.1)	0.33	.72
RBS‐R total score	*n* = 19	*n* = 62	*n* = 23		
Baseline	30.90 (17.30)	28.60 (18.40)	50.90 (24.90)	10.89	<.001
Age (months)	46.10 (7.74)	50.50 (10.40)	49.90 (11.80)	0.60	.55
NVIQ	55.60 (16.2)	63.20 (22.5)	66 (20.5)	1.01	.35

Based on reliable‐change index. ADOS‐2, Autism Diagnostic Observations Schedule‐2; CSS, calibrated severity score; NVIQ, nonverbal IQ; BOSCC, Brief Observation of Social Communication Change; RBS‐R, Repetitive Behaviour Scale‐Revised.

**Table 4:** Baseline characteristics by instruments and individual‐trajectory group

	Individual‐trajectory group			ANOVA	
	Increasing	Stable	Decreasing	*F*	*p*
ADOS2CSSRRB	*n* = 16	*n* = 65	*n* = 53		
Baseline score	5.44 (1.36)	7.68 (0.75)	9.26 (0.66)	148.8	<.001
Age (months)	50.80 (9.33)	49.8 (9.44)	47.2 (11.30)	1.22	.30
NVIQ	65.60 (22.30)	58.40 (19.10)	63.60 (22.30)	1.31	.27
BOSCC‐RRB	*n* = 19	*n* = 67	*n* = 45		
Baseline	10.50 (2.91)	9.96 (3.89)	8.01 (3.03)	5.29	<.01
Age (months)	46.40 (9.89)	50.40 (10.10)	48.20 (10.60)	1.40	.25
NVIQ	54.70 (14.0)	60.50 (22.40)	64.80 (19.50)	1.71	.19
BOSCC‐RRB (MV)	*n* = 16	*n* = 41	*n* = 29		
Baseline score	13.8 (2.81)	11.9 (3.23)	8.02 (2.83)	22.93	<.001
Age (months)	45.3 (10.3)	49.8 (10.3)	48.6 (9.89)	1.10	.34
NVIQ	52.3 (12.5)	49.5 (16.1)	55.2 (10.5)	1.45	.24
RBS‐R total score	*n* = 36	*n* = 68	*n* = 24		
Baseline score	28.50 (15.40)	29.0 (18.70)	51.0 (24.30)	12.77	<.001
Age (months)	49.70 (8.69)	48.50 (10.90)	49.80 (11.60)	0.26	.77
NVIQ	56.10 (20.90)	62.40 (20.30)	65.30 (20.20)	1.72	.18

Based on clustering standardised slopes resulting from linear mixed models. ADOS‐2, Autism Diagnostic Observations Schedule‐2; CSS, calibrated severity score; NVIQ, nonverbal IQ; BOSCC, Brief Observation of Social Communication Change; RBS‐R, Repetitive Behaviour Scale‐Revised.

No differences in baseline age or NVIQ were observed, except for higher NVIQ in the decreasing group for RBS‐R CB.

## Discussion

Measurement issues regarding the RRB symptom domain of ASD have only recently come to attention, despite the clinical and scientific need to reliably measure change in RRB symptoms during intervention and longitudinal assessments (Scahill et al., 2015; Sturm et al., 2022; Uljarević et al., 2023). To support an informed choice of available RRB measures, we examined different RRB measures (ADOS‐2, BOSCC, RBS‐R) characterised by differing sources of information (i.e. parent report vs. behavioural observation) in a sample of preschoolers with ASD concerning change sensitivity as well as their ability to capture individual developmental trajectories in preschoolers.

As hypothesised, our findings suggest varying change sensitivity between instruments.

Absolute change in RRB over 14 months using LMM was detected by ADOS‐2‐CSS‐RRB and RBS‐R RSM. The measures' capability to capture nominal change is important especially for intervention studies and research with trans‐diagnostic samples when time effects between groups are of interest. The large heterogeneity in RRB change over 14 months between participants may explain the null finding of longitudinal mean effects in BOSCC‐RRB and RBS‐R total and other domain scores. The ADOS‐2‐CSS‐RRB, which is tailored to varying developmental stages via module choice, and the RBS‐R RSM subscale may capture preschool specific ASD‐related RRB more adequately, since higher levels of RSM behaviours are expected in younger age (Jasim & Perry, 2023). The contrasting time effects in the RBS‐R RSM subscale versus ADOS‐2‐CSS‐RRB may reflect the circumstances of assessment of the ADOS‐2, which, as an interaction with an unknown person, may elicit more stereotyped behaviour. Replicating previous data (e.g. Antezana et al., 2019; Esbensen, Seltzer, Lam, & Bodfish, 2009), younger children showed more RRB, and boys showed higher levels of RSM behaviour.

As expected, we found high variability of individual RRB symptom trajectories as measured by ADOS‐2‐CSS‐RRB, BOSCC‐RRB and RBS‐R total and subscales, except RBS‐R SI due to the rare occurrence of self‐injury in our sample, which is consistent with previous findings (Kästel et al., 2021).

The RBS‐R total score and subscales IS, RSM and CB detected significantly more participants showing reliable change in RRB (reliable increase or decrease measured by the RCI) than the ADOS‐2‐CSS‐RRB and BOSCC‐RRB. While a three‐group solution (reliable increase, no reliable change, reliable decrease) is inherent to the RCI approach, using the dimensional k‐means algorithm other cluster solutions were also possible. Still, we confirmed three distinct trajectory subgroups (increasing, stable, decreasing) with meaningful sample sizes for ADOS‐2‐CSS‐RRB, the BOSCC‐RRB and the RBS‐R. Although the ADOS‐2 was not designed to capture change, one study using clustering methodology found two RRB severity change groups (Franchini et al., 2023), while another study, using the RCI approach, found three RRB‐trajectory groups (Waizbard‐Bartov et al., 2022). Since both studies were conducted in preschool to school‐aged samples, our results add evidence to three RRB observable change courses during early childhood using behavioural observation (ADOS‐2, BOSCC) and parent report (RBS‐R).

Taken together, the RBS‐R was the most change sensitive measure in this sample of preschoolers with ASD. Variation in change sensitivity across instruments was accompanied by differences in internal consistency, inter‐rater reliability (ADOS‐2, BOSCC) and convergent validity across instruments. The ADOS‐2‐RRB‐CSS demonstrated low and generally unacceptable levels of internal consistency, inter‐rater reliability and sensitivity to change. Similarly, the BOSCC‐RRB showed a moderate level of internal consistency, an acceptable level of inter‐rater reliability and a low sensitivity to change. Contrastingly, the RBS‐R and its subscales showed high levels of internal consistency and acceptable sensitivity to change, except the RBS‐R SI subscale. Convergent validity with the ADI‐R‐RRB was moderate (ADOS‐2‐RRB‐CSS, RBS‐R). This may reflect differences in construct validity between measures. Factor analytic research on RRB is highly suggestive of the multidimensionality of the construct (Uljarević et al., 2023). In line with this reasoning, the ADOS‐2 and BOSCC measure a much smaller range of the varieties of RRB found in preschoolers with ASD than the RBS‐R. Both direct observation instruments only capture RRB elicited during brief structured assessment (ADOS‐2: max. 60 min; BOSCC: 12 min). Consistently, most participants were assigned to differing change groups depending on the implemented instrument.

As a comprehensive, multidimensional caregiver report tool with a low burden of assessment, the RBS‐R may account best for preschool developmental changes that are not captured by diagnostic and direct observation tools like the ADOS‐2‐RRB or BOSCC‐RRB. Not only do they share a higher burden of assessment, but they also do not reliably measure RRB (Grzadzinski et al., 2016; Janvier, Choi, Klein, Lord, & Kim, 2022), which may reflect lower item quantity, lower range of scores and the brief observation time. Still, the RBS‐R has a disadvantage within the context of psychosocial intervention studies, since it cannot be fully blinded within psychosocial intervention studies. Future research may examine the use of the ADI‐R‐RRB subscale, which also allows blinded administering, for evaluating change in RRB.

### Baseline severity and change course

In exploratory comparisons of the reliable‐change and individual‐trajectory groups regarding possible differences in baseline characteristics, we observed different patterns of baseline RRB severity among the groups, both within and between measures. In the RCI‐based reliable‐change groups, a consistent pattern emerged across all measures: The reliable‐increase group had the lowest baseline RRB severity, the no‐reliable‐change group showed medium, and the reliable‐decrease group the highest baseline RRB symptom severity. In the LMM‐based individual‐trajectory groups, this pattern was also observed for the ADOS‐2‐CSS‐RRB and the RBS‐R total and subscale scores, but not for the BOSCC‐RRB. Here, the BOSCC‐RRB decreasing individual‐trajectory group had the lowest baseline RRB severity compared to the increasing and stable trajectory groups. This finding was also observed in the subsample of minimally verbal children; thus, it cannot be explained by language abilities. The specific BOSCC‐RRB baseline severity/trajectory pattern might reflect a lack to capture RRB typically occurring during later developmental stages (Jasim & Perry, 2023). This interpretation is supported by the negative correlations of the BOSCC‐RRB with the RBS‐R subscales IS and CB in our sample. Future research on the BOSCC‐RRB should be conducted additionally using the recently developed version of the BOSCC that offers modules for children with phrase or fluent speech (Byrne et al., 2024) and may allow more adequate assessment of higher age appropriate RRB.

Previous results on baseline symptom severity of RRB‐trajectory groups in preschoolers and school‐aged children have been inconsistent. Some studies have found high baseline symptom severity in stable and less severe symptoms in decreasing (‘improving’) ADOS(‐2)‐derived trajectories both in overall ASD symptomatology (Szatmari et al., 2015) as well as in RRB symptoms (Franchini et al., 2023; Waizbard‐Bartov et al., 2022). Another study found BOSCC‐RRB trajectories to be independent of symptom severity (Grzadzinski et al., 2023). Our results add evidence to the notion that the individual RRB trajectory in preschoolers may be related to baseline symptom severity, albeit in another direction than observed in previous studies.

Across all measures except RBS‐R CB, no differences in baseline age or NVIQ were observed. Previous studies have found mixed results regarding developmental child characteristics: No IQ‐related difference in ADOS‐2 trajectories has been reported (Waizbard‐Bartov et al., 2022), but also increasing or stable high ADOS and BOSCC‐RRB trajectories in children with more developmental difficulties (Franchini et al., 2023; Grzadzinski et al., 2023). Further research on developmental predictors of RRB trajectory is therefore needed.

### Limitations

Although we were able to describe change sensitivity and individual developmental trajectories in commonly used measures using a multimodal approach, a longer observation time span would allow for more elaborate modelling techniques. In addition, we did not compare single items or the underlying factor structures of RRB between and across measures. Further research may aim to identify item combinations with the highest change sensitivity and compare the results to those of other factor analyses.

### Implications for the use of the different instruments

To our knowledge, this is the most comprehensive study in preschoolers to date comparing change sensitivity of three frequently used instruments assessing RRB in young children with ASD over approximately 1 year. Our study showed that the RBS‐R is more sensitive to detecting change in RRB compared to ADOS‐2‐CSS‐RRB and BOSCC‐RRB (minimally verbal version). We found highly variable RRB trajectories in preschoolers with ASD, resulting in three robust distinct trajectory groups using two different methods. However, the overlap of trajectory groups between instruments was moderate, reflecting differing construct validity between measures.

Low internal consistency and inter‐rater reliability of the ADOS‐2 and BOSCC‐RRB subscales call for caution when capturing RRB. They are not suited to capture developmental changes. Our results show that the RBS‐R and its subscales account best for developmental changes in RRB in the preschool age; thus, it can be recommended for longitudinal developmental research or randomised controlled trials. However, it does not allow blinding in psychosocial intervention studies. Ideally, a multimodal approach of assessment using different sources of information should be considered for detailed description of RRB development.

## Ethical considerations

Written informed consent was obtained from the parents after ethical approval by the ethical committees of the medical faculties of the study sites Frankfurt (April 4th, 2018; No. 10/18), Würzburg (June 6th, 2018, No. 102/18_z‐sc), Augsburg (June 26th, 2018; No. 18‐372) and Dresden (January 21st, 2019; No. 41710218).

**Key points**

- Assessment of RRB in preschoolers with ASD is done by different measures using direct behavioural observation or caregiver report questionnaires.
- Comparing change sensitivity across ADOS‐2‐CSS‐RRB, BOSCC‐RRB and RBS‐R, the RBS‐R showed superior change sensitivity combined with overall superior psychometric properties. It can be recommended for detailed description of developmental RRB trajectories.
- ADOS‐2‐CSS‐RRB and BOSCC‐RRB show low internal consistency and inter‐rater reliability as well as sensitivity to change and are not recommended for capturing RRB change.
- RRB trajectories are highly variable and potentially dependent on baseline severity.

## Supporting information


**Table S1.** Weeks since baseline (T1) per instrument and follow up time point.
**Appendix S1.** Procedure for establishing the intra‐class‐correlation (ICC).
**Appendix S2.** Procedure for establishing the Reliable Change Index (RCI)
**Table S2.** Summary statistics of the longitudinally assessed RRB measures and subscales per time point.
**Table S3.** Reliable change group per instrument and time point based on RCI.
**Table S4.** Overlap between reliable change group assignments by ADOS‐2‐CSS‐RRB and BOSCC‐RRB.
**Table S5.** Overlap between reliable change group assignments by ADOS‐2‐CSS‐RRB and RBS‐R total score.
**Table S6.** Overlap between reliable change group assignments by BOSCC‐RRB and RBS‐R total score.
**Table S7.** Beta‐estimates and variance components of random intercept, random slope models predicting RRB‐scores.
**Table S8.** Beta‐estimates and variance components of random intercept models predicting scores of the RBS‐R subscales.
**Figure S1.** BIC for cluster solution per RRB measure.
**Table S9.** BIC per RRB measurement and number of clusters.
**Table 10.** Range of cluster N per RRB measurement and number of clusters.
**Table S11.** Baseline characteristics by Repetitive Behavior scale‐Revised subscale and individual change group.
**Table S12.** Overlap between individual trajectory group assignments by ADOS‐2‐CSS‐RRB and BOSCC‐RRB.
**Table S13.** Overlap between individual trajectory group assignments by ADOS‐2‐CSS‐RRB and RBS‐R total score.
**Table S14.** Overlap between individual trajectory group assignments by ADOS‐2‐CSS‐RRB and BOSCC‐RRB.
**Table S15.** Baseline characteristics by Repetitive Behaviour Scale‐ Revised subscale and reliable change group.
**Table S16.** Results of reliable change group comparisons of baseline score.
**Table S17.** Results of individual trajectory group comparisons of baseline score.

## Data Availability

The data supporting the findings of this study are available from the corresponding author upon reasonable request.
