# Supplementary material for: Capturing change in restricted and repetitive behaviour in preschoolers with ASD: A comparison of direct behavioural observation and parent report
Source: J Child Psychol Psychiatry. 2025 Jul 9;66(11):1736–47. doi: 10.1111/jcpp.70009 (PMC12571943; doi:10.1111/jcpp.70009)
Supplement: Supplementary file 1 — Table S1. Weeks since baseline (T1) per instrument and follow up time point. Appendix S1. Procedure for establishing the intra‐class‐correlation (ICC). Appendix S2. Procedure for establishing the Reliable Change Index (RCI) Table S2. Summary statistics of the longitudinally assessed RRB measures and subscales per time point. Table S3. Reliable change group per instrument and time point based on RCI. Table S4. Overlap between reliable change group assignments by ADOS‐2‐CSS‐RRB and BOSCC‐RRB. Table S5. Overlap between reliable change group assignments by ADOS‐2‐CSS‐RRB and RBS‐R total score. Table S6. Overlap between reliable change group assignments by BOSCC‐RRB and RBS‐R total score. Table S7. Beta‐estimates and variance components of random intercept, random slope models predicting RRB‐scores. Table S8. Beta‐estimates and variance components of random intercept models predicting scores of the RBS‐R subscales. Figure S1. BIC for cluster solution per RRB measure. Table S9. BIC per RRB measurement and number of clusters. Table 10. Range of cluster N per RRB measurement and number of clusters. Table S11. Baseline characteristics by Repetitive Behavior scale‐Revised subscale and individual change group. Table S12. Overlap between individual trajectory group assignments by ADOS‐2‐CSS‐RRB and BOSCC‐RRB. Table S13. Overlap between individual trajectory group assignments by ADOS‐2‐CSS‐RRB and RBS‐R total score. Table S14. Overlap between individual trajectory group assignments by ADOS‐2‐CSS‐RRB and BOSCC‐RRB. Table S15. Baseline characteristics by Repetitive Behaviour Scale‐ Revised subscale and reliable change group. Table S16. Results of reliable change group comparisons of baseline score. Table S17. Results of individual trajectory group comparisons of baseline score. [file JCPP-66-1736-s001.docx]

**Supporting information for**

**Capturing change in restricted and repetitive behaviour in preschoolers with ASD – a comparison of direct behavioural observation and parent report**

Authors: Naisan Raji, Janina Kitzerow-Cleven, Ziyon Kim, Solvejg K. Kleber, Leonie Polzer, Christian Lemler, Melanie Ring, Regina Taurines, Julia Geißler, Ulrike Fröhlich, Michele Noterdaeme, Nico Bast, Christine M. Freitag

[Table S1. Weeks since baseline (T1) per instrument and follow up time point. 3](#_Toc171089133)

[Appendix S1. Procedure for establishing the intra-class-correlation (ICC) 3](#_Toc171089134)

[Appendix S2. Procedure for establishing the Reliable Change Index (RCI) 4](#_Toc171089135)

[Table S2. Summary statistics of the longitudinally assessed RRB measures and subscales per time point. 5](#_Toc171089136)

[Table S3. Reliable change group per instrument and time point based on RCI. 6](#_Toc171089137)

[Table S4. Overlap between reliable change group assignments by ADOS-2-CSS-RRB and BOSCC-RRB. 7](#_Toc171089138)

[Table S5. Overlap between reliable change group assignments by ADOS-2-CSS-RRB and RBS-R total score. 7](#_Toc171089139)

[Table S6. Overlap between reliable change group assignments by BOSCC-RRB and RBS-R total score. 8](#_Toc171089140)

[Table S7. Beta-estimates and variance components of random intercept, random slope models predicting RRB-scores. 9](#_Toc171089141)

[Table S8. Beta-estimates and variance components of random intercept models predicting scores of the RBS-R subscales. 10](#_Toc171089142)

[Figure S1. BIC for cluster solution per RRB measure. 11](#_Toc171089143)

[Table S9. BIC per RRB measurement and number of clusters 12](#_Toc171089144)

[Table 10. Range of cluster N per RRB measurement and number of clusters 12](#_Toc171089145)

[Table S11. Baseline characteristics by Repetitive Behavior scale-Revised subscale and individual change group. 13](#_Toc171089146)

[Table S12. Overlap between individual trajectory group assignments by ADOS-2-CSS-RRB and BOSCC-RRB. 14](#_Toc171089147)

[Table S13. Overlap between individual trajectory group assignments by ADOS-2-CSS-RRB and RBS-R total score. 14](#_Toc171089148)

[Table S14. Overlap between individual trajectory group assignments by ADOS-2-CSS-RRB and BOSCC-RRB. 15](#_Toc171089149)

[Table S15. Baseline characteristics by Repetitive Behaviour Scale- Revised subscale and reliable change group. 16](#_Toc171089150)

[Table S16. Results of reliable change group comparisons of baseline score. 17](#_Toc171089151)

[Table S17. Results of individual trajectory group comparisons of baseline score. 19](#_Toc171089152)

[References 21](#_Toc171089153)

# Table S1. Weeks since baseline (T1) per instrument and follow up time point.

|  | T4 | T6 |
| --- | --- | --- |
| ADOS-2-CSS-RRB | 31.82 (5.55) | 61.42 (6.64) |
| BOSCC-RRB | 30.63 (5.58) | 60.06 (6.25) |
| RBS-R | 29.58 (5.99) | 58.58 (7.68) |

ADOS-2, Autism Diagnostic Observation Schedule-2; CSS, Calibrated Severity Score; BOSCC, Brief Observation of Social Communication Change; RBS-R, Repetitive Behaviour Scale-Revised.

# Appendix S1. Procedure for establishing the intra-class-correlation (ICC)

For each multiply rated video, two ratings by two different raters were randomly selected, resulting in a data set consisting of pairs of independent ratings for the same video. To this data set, the following linear mixed model was applied (Shrout & Fleiss, 1979):

$$x_{ij}=\mu+b_{j}+\epsilon_{ij},$$

with

$x_{ij}$ = rating of the video $j$ rated by one of the two randomly selected raters $i$

$\mu$ = unknown true population mean score

$b_{j}$ = unknown difference between the subject’s true score and the population mean, modelled as random effect with mean $0$ and variance $\sigma_{b}^{2}$

$\epsilon_{ij}$ = random deviation from the subject’s true score that was introduced by the rater, modelled with mean $0$ and variance $\sigma_{\epsilon}^{2}$.

The intra-class correlation coefficient (ICC) was subsequently calculated as

$ICC=\sqrt{\frac{\sigma_{b}^{2}}{\sigma_{b}^{2}+\sigma_{\epsilon}^{2}}}$.

Confidence intervals of the ICCs were obtained using the jack-knife method together with Fisher’s Z-transformation (Feng, Svetnik, Coimbra, & Baumgartner, 2014).

# Appendix S2. Procedure for establishing the Reliable Change Index (RCI)

The RCI was established using following formula (Jacobson & Truax, 1991):

$$\text{RCI = }\frac{\text{x}_{\text{2}}- \text{x}_{\text{1}}}{\sqrt{\text{2 (}\text{SD}\sqrt{1- r_{\text{xx}}})^{2}}}$$

with

*x_1_ =* a subject's pretest score

*x_2_ =* that same subject's posttest score

*SD =* standard deviation at pretest

*r_xx_ =* test-retest reliability

Test-retest reliability coefficients $r_{\text{xx}}$ were derived from the following publications:

*ADOS-2-CSS-RRB*. Derived from Janvier et al. (2022)

*BOSCC-RRB*. Derived from Grzadzinski et al. (2016)

*RBS-R*. To date, there is no test-retest-reliability for the RBS-R available, which is why we used the internal consistency of the RBS-R total and subscales as reported by Kästel et al. (2021).

# Table S2. Summary statistics of the longitudinally assessed RRB measures and subscales per time point.

|  | T1 | | T4 | | T6 | |
| --- | --- | --- | --- | --- | --- | --- |
|  | N | M (SD) | n | M (SD) | n | M (SD) |
| ADOS-2-CSS-RRB | 134 | 8.04 (1.46) | 118 | 8.27 (1.23) | 114 | 8.31 (1.28) |
| BOSCC-RRB | 129 | 9.37 (3.60) | 120 | 10.01 (3.87) | 118 | 9.6 (4.14) |
| RBS-R total | 119 | 33.31 (20.99) | 115 | 33.25 (21.32) | 113 | 33.55 (21.85) |
| RBS-R IS | 119 | 16.83 (10.95) | 116 | 17.46 (11.22) | 112 | 17.61 (11.20) |
| RBS-R RSM | 120 | 9.13 (5.80) | 115 | 8.64 (5.97) | 113 | 8.43 (5.99) |
| RBS-R SI | 123 | 1.89 (3.26) | 116 | 2.05 (2.65) | 114 | 2.13 (3.13) |
| RBS-R CB | 120 | 5.13 (5.11) | 116 | 5.44 (5.12) | 114 | 5.35 (4.97) |
| ADOS-2, Autism Diagnostic Observations Schedule-2; CSS, calibrated severity score; BOSCC, Brief Observation of Social Communication Change; RBS-R, Repetitive Behaviour Scale-Revised; IS, insistence on sameness; RSM, repetitive sensorimotor movements; SI, self-injurious behaviour; CB, compulsive behaviour. | | | | | | |

# Table S3. Reliable change group per instrument and time point based on RCI.

|  | T1 to T4 (%) | | | | T4 to T6 (%) | | | | T1 to T6 (%) | | | |
| --- | --- | --- | --- | --- | --- | --- | --- | --- | --- | --- | --- | --- |
|  | n | decrease^1^ | increase^2^ | no change^3^ | n | decrease | increase | no change | n | decrease | increase | no change |
| ADOS‑2‑CSS‑RRB | 118 | 0 | 7 (5.93) | 111 (94.07) | 110 | 3 (2.73) | 4 (3.64) | 103 (93.64) | 114 | 2 (1.75) | 9 (7.89) | 103 (90.35) |
| BOSCC‑RRB | 118 | 5 (4.24) | 10 (8.47) | 103 (87.29) | 114 | 6 (5.26) | 4 (3.51) | 104 (91.23) | 117 | 6 (5.13) | 8 (6.84) | 103 (88.03) |
| BOSCC‑RRB (MV) | 85 | 4 (4.71) | 10 (11.76) | 71 (83.53) | 74 | 6 (8.11) | 3 (4.05) | 65 (87.84) | 76 | 5 (6.58) | 9 (11.84) | 62 (81.58) |
| RBS-R total | 107 | 18 (16.82) | 16(14.95) | 73 (68.22) | 107 | 16 (14.95) | 12 (11.21) | 79 (73.82) | 104 | 23 (22.12) | 19 (18.27) | 62 (59.62) |
| RBS-R IS | 108 | 15(13.89) | 14(12.96) | 79 (73.15) | 107 | 12 (11.21) | 9 (8.41) | 86 (80.37) | 104 | 17 (16.35) | 14 (13.46) | 73 (70.19) |
| RBS-R RSM | 108 | 10 (9.26) | 5 (4.63) | 93 (86.11) | 107 | 8 (7.48) | 9 (8.41) | 90 (84.11) | 105 | 16 (15.24) | 6 (5.71) | 83 (79.05) |
| RBS-R SI | 112 | 4 (3.57) | 3 (2.68) | 93 (83.04) | 109 | 4 (3.67) | 6 (5.5) | 99 (90.83) | 109 | 5 (4.59) | 6 (5.5) | 98 (89.91) |
| RBS-R CB | 109 | 5 (4.59) | 9 (8.26) | 95 (87.16) | 109 | 6 (5.5) | 1 (0.92) | 102 (93.58) | 106 | 12 (11.32) | 7 (6.6) | 87 (82.1) |
| ^1^Reliable symptom decrease, i.e. lower scoring. ^2^Reliable symptom increase, i.e. higher scoring. ^3^No reliable change. ADOS-2, Autism Diagnostic Observation Schedule-2; CSS, Calibrated Severity Score; BOSCC, Brief Observation of Social Communication Change; MV, minimally verbal; RBS-R, Repetitive Behaviour Scale-Revised; IS, Insistence on sameness; RSM, Repetitive sensorimotor movements; SI, Self-injurious behaviour; CB, Compulsive behaviour. | | | | | | | | | | | | |

# Table S4. Overlap between reliable change group assignments by ADOS-2-CSS-RRB and BOSCC-RRB.

|  |  | BOSCC-RRB | | |
| --- | --- | --- | --- | --- |
|  |  | Reliable symptom increase | No reliable change | Reliable symptom decrease |
| ADOS-2-CSS-RRB | Reliable symptom increase | 0 | 7 (6.2 %) | 2 (1.8 %) |
|  | No reliable change | 8 (7.1 %) | 91 (80.5 %) | 3 (2.7 %) |
|  | Reliable symptom decrease | 0 | 1 (0.9 %) | 1 (0.9 %) |

Reliable change group assignment based on Reliable Change Index (T1-T6). n = 113. ADOS-2, Autism Diagnostic Observation Schedule-2; CSS,

Calibrated Severity Score; BOSCC, Brief Observation of Social Communication Change.

# Table S5. Overlap between reliable change group assignments by ADOS-2-CSS-RRB and RBS-R total score.

|  |  | RBS-R | | |
| --- | --- | --- | --- | --- |
|  |  | Reliable symptom increase | No reliable change | Reliable symptom decrease |
| ADOS-2-CSS-RRB | Reliable symptom increase | 1 (1 %) | 5 (4.9 %) | 2 (2 %) |
|  | No reliable change | 18 (17.6 %) | 55 (53.9 %) | 19 (18.6 %) |
|  | Reliable symptom decrease | 0 | 1 (1 %) | 1 (1 %) |

Reliable change group assignment based on Reliable Change Index (T1-T6). n = 102. ADOS-2, Autism Diagnostic Observation Schedule-2; CSS,

Calibrated Severity Score; RBS-R, Repetitive Behaviour Scale-Revised.

# Table S6. Overlap between reliable change group assignments by BOSCC-RRB and RBS-R total score.

|  |  | RBS-R | | |
| --- | --- | --- | --- | --- |
|  |  | Reliable symptom increase | No reliable change | Reliable symptom decrease |
| BOSCC-RRB | Reliable symptom increase | 0 | 5 (4.8 %) | 1 (1 %) |
|  | No reliable change | 19 (18.3 %) | 53 (51 %) | 20 (19.2 %) |
|  | Reliable symptom decrease | 0 | 4 (3.8 %) | 2 (1.9 %) |

Reliable change group assignment based on Reliable Change Index (T1-T6). n = 104. BOSCC, Brief Observation of Social Communication

Change; RBS-R, Repetitive Behaviour Scale-Revised.

# Table S7. Beta-estimates and variance components of random intercept, random slope models predicting RRB-scores.

|  | ADOS-2-RRB-CSS | | BOSCC-RRB | | BOSCC-RRB (MV) | | RBS-R total | |
| --- | --- | --- | --- | --- | --- | --- | --- | --- |
| Fixed effects | Estimate (SE) | *p* | Estimate (SE) | *p* | Estimate (SE) | *p* | Estimate (SE) | *p* |
| Intercept | 7.71 (0.28) | <0.001 | 9.20 (0.74) | <0.001 | 8.83 (0.86) | <0.001 | 51.61 (5.40) | <0.001 |
| Time point | 0.15 (0.07) | 0.03 | 0.06 (0.14) | 0.66 | 0.16 (0.18) | 0.38 | -0.51 (0.75) | 0.50 |
| Age | -0.02 (0.01) | 0.03 | -0.06 (0.02) | 0.01 | -0.06 (0.03) | 0.06 | -0.13 (0.17) | 0.46 |
| Gender | -0.27 (0.23) | 0.24 | -0.19 (0.61) | 0.75 | -0.35 (0.70) | 0.62 | -8.1 (4.36) | 0.07 |
| ISCED | 0.09 (0.09) | 0.30 | 0.15 (0.25) | 0.53 | 0.33 (0.29) | 0.27 | -5.1 (1.78) | <0.01 |
| NVIQ | -0.001 (0.004) | 0.82 | -0.11 (0.01) | <0.001 | -0.12 (0.02) | <0.001 | 0.11 (0.09) | 0.24 |
| Site | 0.18 (0.08) | 0.03 | -0.05 (0.22) | 0.79 | -0.22 (0.26) | 0.41 | -2.38 (1.55) | 0.13 |
| Random parts | Variance component | SD | Variance component | SD | Variance component | SD | Variance component | SD |
| Intercept | 0.78 | 0.88 | 5.13 | 2.26 | 5.65 | 2.38 | 352.01 | 18.76 |
| Time point | 0.03 | 0.17 | 0.36 | 0.6 | 0.60 | 0.77 | 28.32 | 5.32 |
| Residual | 1.03 | 1.02 | 4.11 | 2.03 | 4.35 | 2.09 | 70.24 | 8.38 |

ADOS-2, Autism Diagnostic Observation Schedule-2; CSS, Calibrated Severity Score; BOSCC, Brief Observation of Social Communication Change; RBS-R,

Repetitive Behaviour Scale-Revised.

# Table S8. Beta-estimates and variance components of random intercept models predicting scores of the RBS-R subscales.

|  | RBS-R IS | | RBS-R RSM | | RBS-R SI | | RBS-R CB | |
| --- | --- | --- | --- | --- | --- | --- | --- | --- |
| Fixed effects | Estimate (SE) | *p* | Estimate (SE) | *p* | Estimate (SE) | *p* | Estimate (SE) | *p* |
| Intercept | 24.88 (2.77) | <.001 | 13.42 (1.46) | <.001 | 3.28 (0.72) | <.001 | 9.35 (1.23) | <.001 |
| Time point | 0.10 (0.40) | 0.80 | -0.55 (0.22) | 0.01 | 0.07 (0.16) | 0.68 | 0.01 (0.20) | 0.95 |
| Age | 0.001 (0.09) | 0.99 | -0.05 (0.05) | 0.27 | -0.03 (0.02) | 0.13 | -0.05 (0.04) | 0.24 |
| Gender | -2.63 (2.23) | 0.24 | -3.32 (1.18) | 0.01 | -0.54 (0.57) | 0.35 | -1.31 (0.99) | 0.19 |
| ISCED | -2.47 (0.91) | 0.01 | -1.02 (0.48) | 0.04 | -0.37 (0.23) | 0.12 | -1.13 (0.40) | 0.01 |
| NVIQ | 0.14 (0.04) | <0.01 | -0.05 (0.02) | 0.04 | -0.01 (0.01) | 0.39 | 0.03 (0.02) | 0.14 |
| Site | -0.58 (0.79) | 0.47 | -0.71 (0.42) | 0.09 | -0.19 (0.20) | 0.34 | -0.79 (0.35) | 0.03 |
| Random parts | Variance component | SD | Variance component | SD | Variance component | SD | Variance component | SD |
| Intercept | 89.87 | 9.48 | 24.54 | 4.95 | 7.39 | 2.72 | 18.62 | 4.32 |
| Time point | 7.97 | 2.82 | 0.90 | 0.95 | 1.42 | 1.19 | 1.83 | 1.35 |
| Residual | 20.33 | 4.51 | 8.49 | 2.91 | 2.91 | 1.71 | 5.81 | 2.41 |

RBS-R, Repetitive Behaviour Scale-Revised; IS, Insistence on sameness; RSM, Repetitive sensorimotor movements; SI, Self-injurious behaviour; CB,

Compulsive behaviour.

# Figure S1. BIC for cluster solution per RRB measure.


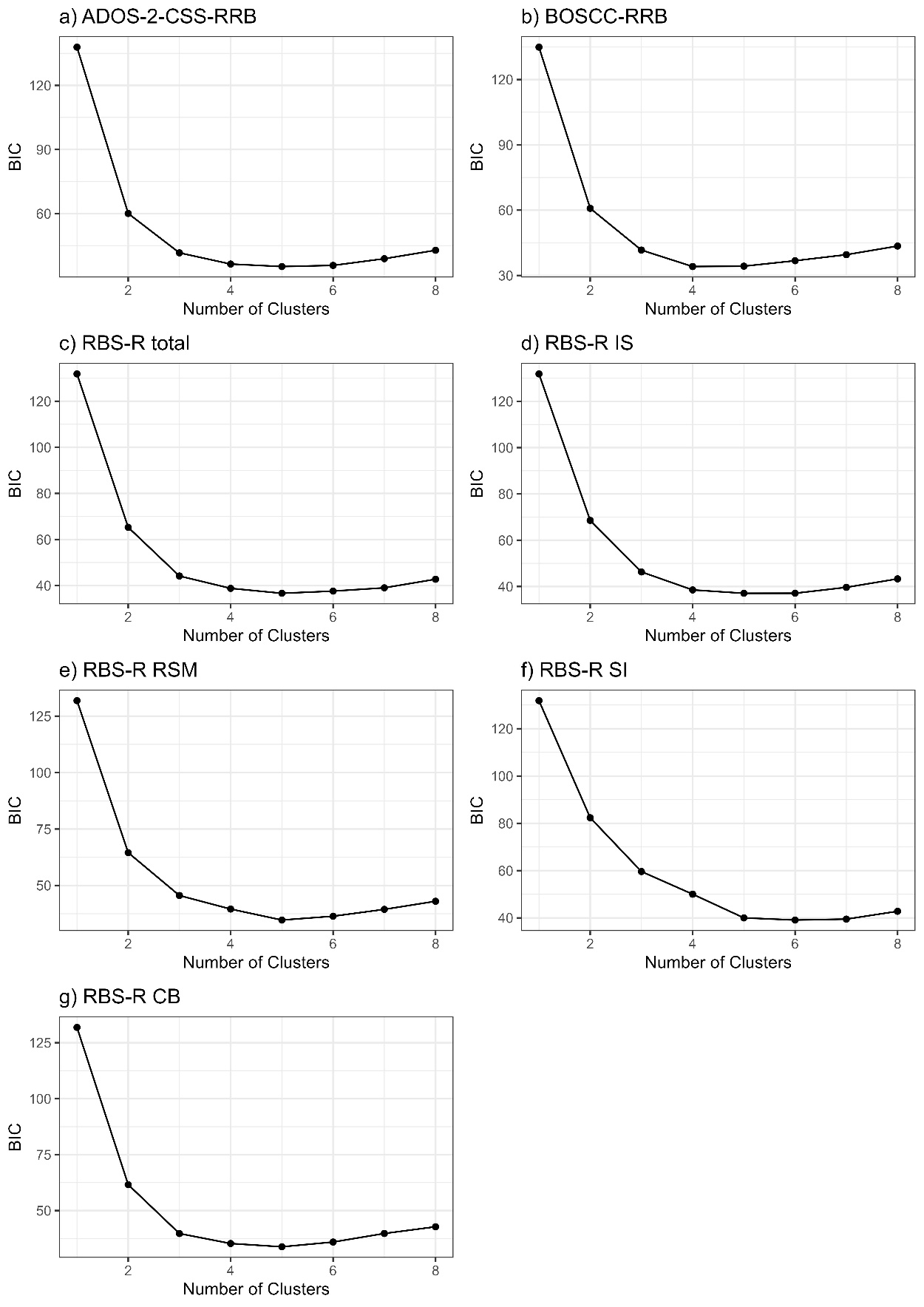


ADOS-2, Autism Diagnostic Observation Schedule-2; CSS, Calibrated Severity Score; BOSCC, Brief Observation of Social Communication Change; RBS-R, Repetitive Behaviour Scale-Revised; IS, Insistence on sameness; RSM, Repetitive sensorimotor movements; SI, Self-injurious behaviour; CB, Compulsive behaviour.

# Table S9. BIC per RRB measurement and number of clusters

|  | Number of clusters | | | | | | | |
| --- | --- | --- | --- | --- | --- | --- | --- | --- |
|  | 1 | 2 | 3 | 4 | 5 | 6 | 7 | 8 |
| ADOS‑2‑CSS‑RRB | 137.90 | 60.04 | 41.61 | 36.36 | 35.20 | 35.73 | 38.87 | 42.82 |
| BOSCC‑RRB | 134.88 | 60.83 | 41.65 | 34.10 | 34.26 | 36.77 | 39.54 | 43.51 |
| RBS-R total | 131.85 | 65.28 | 44.16 | 38.80 | 36.67 | 37.61 | 39.04 | 42.76 |
| RBS-R IS | 131.85 | 68.54 | 46.30 | 38.52 | 37.07 | 37.09 | 39.64 | 43.30 |
| RBS-R RSM | 131.85 | 64.54 | 45.56 | 39.56 | 34.70 | 36.39 | 39.43 | 43.01 |
| RBS-R SI | 131.85 | 82.35 | 59.61 | 50.03 | 40.02 | 39.10 | 39.49 | 42.80 |
| RBS-R CB | 131.85 | 61.59 | 39.74 | 35.28 | 33.82 | 35.94 | 39.77 | 42.76 |

ADOS-2, Autism Diagnostic Observation Schedule-2; CSS, Calibrated Severity Score; BOSCC, Brief Observation of Social Communication Change; RBS-R, Repetitive Behaviour Scale-Revised; IS, Insistence on sameness; RSM, Repetitive sensorimotor movements; SI, Self-injurious behaviour; CB, Compulsive behaviour.

# Table 10. Range of cluster N per RRB measurement and number of clusters

|  | Number of clusters | | | | | | | |
| --- | --- | --- | --- | --- | --- | --- | --- | --- |
|  | 1 | 2 | 3 | 4 | 5 | 6 | 7 | 8 |
| ADOS‑2‑CSS-RRB | 134 | 54-80 | 16-65 | 10-46 | 1-46 | 1-35 | 1-32 | 1-32 |
| BOSCC‑RRB | 131 | 52-79 | 19-67 | 12-54 | 11-39 | 1-39 | 3-34 | 1-33 |
| RBS-R total | 128 | 54-74 | 24-68 | 7-60 | 7-48 | 5-38 | 2-38 | 2-26 |
| RBS-R IS | 128 | 60-68 | 12-80 | 4-57 | 4-57 | 3-39 | 2-32 | 2-32 |
| RBS-R RSM | 128 | 42-86 | 26-70 | 9-50 | 3-50 | 3-42 | 3-29 | 2-29 |
| RBS-R SI | 128 | 8-120 | 7-113 | 5-85 | 1-83 | 1-71 | 1-61 | 1-60 |
| RBS-R CB | 128 | 33-95 | 29-65 | 8-62 | 5-61 | 5-49 | 5-29 | 5-29 |

ADOS-2, Autism Diagnostic Observation Schedule-2; CSS, Calibrated Severity Score; BOSCC, Brief Observation of Social Communication Change; RBS-R, Repetitive Behaviour Scale-Revised; IS, Insistence on sameness; RSM, Repetitive sensorimotor movements; SI, Self-injurious behaviour; CB, Compulsive behaviour

# Table S11. Baseline characteristics by Repetitive Behavior scale-Revised subscale and individual-trajectory group.

|  |  | Individual-trajectory group | | | ANOVA | |
| --- | --- | --- | --- | --- | --- | --- |
|  |  | increasing | stable | decreasing | *F* | *P* |
| RBS-R IS | | n = 12 | n = 80 | n = 36 |  |  |
|  | Baseline | 17.30 (9.72) | 14.10 (10.70) | 22.60 (9.84) | 7.88 | <.001 |
|  | Age | 45.40 (7.97) | 49 (10.20) | 50.40 (11.50) | 1.02 | .36 |
|  | NVIQ | 54.10 (13.30) | 59.90 (20.50) | 66.40 (21.90) | 2.05 | .13 |
| RBS‑R RSM | | n=32 | n=70 | n=26 |  |  |
|  | Baseline | 5.94 (4.97) | 8.65 (4.88) | 14.1 (5.89) | 17.79 | <.001 |
|  | Age | 49.10 (9.59) | 49.10 (10.40) | 48.90 (11.70) | 0.004 | .99 |
|  | NVIQ | 57.40 (18.50) | 62.90 (20.80) | 61 (22.20) | 0.80 | .45 |
| RBS-R SI | | n=8 | n=113 | n=7 |  |  |
|  | Baseline | 1.71 (1.89) | 1.25 (1.66) | 12 (5.97) | 85.83 | <.001 |
|  | Age | 43.40 (7.63) | 49.50 (10.50) | 48 (11) | 1.37 | .26 |
|  | NVIQ | 54 (11) | 61.50 (21.20) | 64.30 (18.80) | 0.58 | .56 |
| RBS-R CB | | n=34 | n=65 | n=29 |  |  |
|  | Baseline | 3.59 (3.59) | 3.52 (3.87) | 10.10 (5.80) | 25.68 | <.001 |
|  | Age | 49 (9.32) | 49.1 (10.20) | 49.10 (12.20) | 0.001 | .99 |
|  | NVIQ | 55.60 (19.10) | 59.60 (20.60) | 63.80 (28.20) | 5.18 | .01 |

Based on clustering standardised slopes resulting from linear mixed models. RBS-R, Repetitive Behaviour Scale-Revised; IS, insistence on sameness; RSM, repetitive sensorimotor movements; SI, self-injurious behaviour; CB, compulsive behaviour.

# Table S12. Overlap between individual-trajectory group assignments by ADOS-2-CSS-RRB and BOSCC-RRB.

|  |  | BOSCC-RRB | | |
| --- | --- | --- | --- | --- |
|  |  | Increasing | Stable | Decreasing |
| ADOS-2-CSS-RRB | Increasing | 0 | 3 (2.2 %) | 13 (9.9 %) |
|  | Stable | 11 (8.3 %) | 33 (25.2 %) | 19 (14.5 %) |
|  | Decreasing | 8 (6.1 %) | 31 (23.7 %) | 13 (9.9 %) |

Individual trajectory group assignment based on clustering of standardised LMM-slopes. n = 131. ADOS-2, Autism Diagnostic Observation Schedule-2; CSS, Calibrated Severity Score; BOSCC, Brief Observation of Social Communication Change.

# Table S13. Overlap between individual-trajectory group assignments by ADOS-2-CSS-RRB and RBS-R total score.

|  |  | RBS-R | | |
| --- | --- | --- | --- | --- |
|  |  | Increasing | Stable | Decreasing |
| ADOS-2-CSS-RRB | Increasing | 2 (1.6 %) | 9 (7 %) | 5 (3.9 %) |
|  | Stable | 21 (16.4 %) | 29 (22.7 %) | 12 (9.4 %) |
|  | Decreasing | 13 (10.2 %) | 30 (23.4 %) | 7 (5.5 %) |

Individual trajectory group assignment based on clustering of standardised LMM-slopes. n = 128. ADOS-2, Autism Diagnostic Observation Schedule-2; CSS, Calibrated Severity Score; RBS-R, Repetitive Behaviour Scale-Revised.

# Table S14. Overlap between individual-trajectory group assignments by ADOS-2-CSS-RRB and BOSCC-RRB.

|  |  | RBS-R | | |
| --- | --- | --- | --- | --- |
|  |  | Increasing | Stable | Decreasing |
| BOSCC-RRB | Increasing | 4 (3.1 %) | 14 (10.9 %) | 1 (0.8 %) |
|  | Stable | 23 (19 %) | 31 (24.2 %) | 10 (7.8 %) |
|  | Decreasing | 9 (7 %) | 23 (18 %) | 13 (10.2 %) |

Individual trajectory group assignment based on clustering of standardised LMM-slopes. n = 128. BOSCC, Brief Observation of Social Communication Change; RBS-R, Repetitive Behaviour Scale-Revised.

# Table S15. Baseline characteristics by Repetitive Behaviour Scale- Revised subscale and reliable-change group.

|  |  | Reliable change group | | | ANOVA | |
| --- | --- | --- | --- | --- | --- | --- |
|  |  | Reliable symptom increase | No reliable change | Reliable symptom decrease | *F* | *P* |
| RBS-R IS | | n = 14 | n = 73 | n = 17 |  |  |
|  | Baseline | 15.70 (10.20) | 15.10 (10.70) | 27.50 (8.15) | 10.27 | <.001 |
|  | Age | 47 (8.90) | 49.70 (10.20) | 51.60 (12.40) | 0.78 | .47 |
|  | NVIQ | 60.20 (19.70) | 60.80 (21) | 71.20 (22.40) | 1.77 | .18 |
| RBS‑R RSM | | n=6 | n=83 | n=16 |  |  |
|  | Baseline | 5.17 (2.93) | 8.48 (5.59) | 15.10 (5.51) | 11.69 | <.001 |
|  | Age | 50 (8.15) | 49 (10.50) | 51.60 (10.70) | 0.45 | .64 |
|  | NVIQ | 54 (19.9) | 63.7 (20.8) | 59.90 (22.9) | 0.74 | .48 |
| RBS-R SI | | n=6 | n=98 | n=5 |  |  |
|  | Baseline | 1.67 (2.07) | 1.31 (1.78) | 13 (7) | 65.56 | <.001 |
|  | Age | 41.70 (8.16) | 50.10 (10.30) | 51.20 (10.60) | 1.95 | .15 |
|  | NVIQ | 53.50 (12.30) | 62.50 (21.30) | 65.20 (21.50) | 0.58 | .56 |
| RBS-R CB | | n=7 | n=87 | n=12 |  |  |
|  | Baseline | 3.71 (3.30) | 4.01 (3.84) | 14.9 (5.50) | 10.27 | <.001 |
|  | Age | 44.60 (7.91) | 49.7 (10.20) | 49.90 (12.60) | 0.77 | .47 |
|  | NVIQ | 57.30 | 61.10 | 69.20 | 1.77 | .18 |

Based on reliable change index. RBS-R, Repetitive Behaviour Scale-Revised; IS, insistence on sameness; RSM, repetitive sensorimotor movements; SI, self-injurious behaviour; CB, compulsive behaviour.

# Table S16. Results of reliable-change group comparisons of baseline score.

|  |  | Mean difference | 95% CI | *p* |
| --- | --- | --- | --- | --- |
| ADOS-2-CSS-RRB | |  |  |  |
|  | No reliable change vs. reliable increase | 2.73 | 1.65, 3.81 | <.001 |
|  | Reliable decrease vs. reliable increase | 4.06 | 1.63, 6.48 | <.001 |
|  | Reliable decrease vs. no reliable change | 1.33 | -0.89, 3.54 | .33 |
| BOSCC-RRB | |  |  |  |
|  | No reliable change vs. reliable increase | 1.27 | -1.84, 4.39 | .60 |
|  | Reliable decrease vs. reliable increase | 4.73 | 0.14, 9.31 | .04 |
|  | Reliable decrease vs. no reliable change | 3.46 | -0.11, 7.02 | .06 |
| RBS-R total score | |  |  |  |
|  | No reliable change vs. reliable increase | -2.23 | -14.57, 10.11 | .90 |
|  | Reliable decrease vs. reliable increase | 20.02 | 5.43, 34.61 | <.01 |
|  | Reliable decrease vs. no reliable change | 22.25 | 10.76, 33.75 | <.001 |
| RBS-R IS | |  |  |  |
|  | No reliable change vs. reliable increase | -0.58 | -7.69, 6.53 | .98 |
|  | Reliable decrease vs. reliable increase | 11.82 | 3.02, 20.61 | <.01 |
|  | Reliable decrease vs. no reliable change | 12.39 | 5.83, 18.95 | <.001 |
| RBS-R RSM | |  |  |  |
|  | No reliable change vs. reliable increase | 3.32 | -2.19, 8.82 | .33 |
|  | Reliable decrease vs. reliable increase | 9.96 | 3.72, 16.19 | <.001 |
|  | Reliable decrease vs. no reliable change | 6.64 | 3.09, 10.20 | <.001 |
| RBS-R SI | |  |  |  |
|  | No reliable change vs. reliable increase | -0.36 | -2.59, 1.87 | .92 |
|  | Reliable decrease vs. reliable increase | 11.33 | 8.12, 14.54 | <.001 |
|  | Reliable decrease vs. no reliable change | 11.69 | 9.27, 14.12 | <.001 |
|  |  |  |  |  |
| **Table S16** (continued). | |  |  |  |
| RBS-R CB | |  |  |  |
|  | No reliable change vs. reliable increase | 0.29 | -3.47, 4.05 | .98 |
|  | Reliable decrease vs. reliable increase | 11.20 | 6.65, 15.75 | <.001 |
|  | Reliable decrease vs. no reliable change | 10.91 | 7.96, 13.86 | <.001 |

Based on reliable change index. ADOS-2, Autism Diagnostic Observations Schedule-2; CSS, calibrated severity score; BOSCC, Brief Observation of Social Communication Change; RBS-R, Repetitive Behaviour Scale-Revised; IS, insistence on sameness; RSM, repetitive sensorimotor movements; SI, self-injurious behaviour; CB, compulsive behaviour.

# Table S17. Results of individual-trajectory group comparisons of baseline score.

|  |  | Mean difference | 95% CI | *p* |
| --- | --- | --- | --- | --- |
| ADOS-2-CSS-RRB | |  |  |  |
|  | Stable vs. increasing | 2.24 | 1.70, 2.78 | <.001 |
|  | Decreasing vs. increasing | 3.83 | 3.28, 4.38 | <.001 |
|  | Decreasing vs. stable | 1.59 | 1.23, 1.94 | <.001 |
| BOSCC-RRB | |  |  |  |
|  | Stable vs. increasing | -0.54 | -2.69-1.62 | .82 |
|  | Decreasing vs. increasing | -2.49 | -4.76, -0.21 | .03 |
|  | Decreasing vs. stable | -1.95 | -3.56, -0.34 | .01 |
| RBS-R total score | |  |  |  |
|  | Stable vs. increasing | 0.51 | -9.29, 10.31 | .99 |
|  | Decreasing vs. increasing | 22.46 | 10.25, 34.66 | <.001 |
|  | Decreasing vs. stable | 21.95 | 11.01, 32.88 | <.001 |
| RBS-R IS | |  |  |  |
|  | Stable vs. increasing | -3.17 | -11.12, 4.78 | .61 |
|  | Decreasing vs. increasing | 5.35 | -3.18, 13.89- | .30 |
|  | Decreasing vs. stable | 8.52 | 3.41, 13.62 | <.001 |
| RBS-R RSM | |  |  |  |
|  | Stable vs. increasing | 2.72 | 0.01, 5.43 | .05 |
|  | Decreasing vs. increasing | 8.18 | 4.87, 11.50 | <.001 |
|  | Decreasing vs. stable | 5.47 | 2.61, 8.32 | <.001 |
| RBS-R SI | |  |  |  |
|  | Stable vs. increasing | -0.47 | -2.41, 1.48 | .84 |
|  | Decreasing vs. increasing | 10.29 | 7.62, 12.96 | <.001 |
|  | Decreasing vs. stable | 10.75 | 8.80, 12.70 | <.001 |
| **Table S17** (continued). | | | | |
| RBS-R CB | |  |  |  |
|  | Stable vs. increasing | -0.07 | -2.30, 2.15 | .99 |
|  | Decreasing vs. increasing | 6.51 | 3.92, 9.11 | <.001 |
|  | Decreasing vs. stable | 6.59 | 4.27, 8.90 | <.001 |

Based on clustering standardised slopes resulting from linear mixed models.

ADOS-2, Autism Diagnostic Observations Schedule-2; CSS, calibrated severity score;

NVIQ, non-verbal IQ; BOSCC, Brief Observation of Social Communication Change;

RBS-R, Repetitive Behaviour Scale-Revised; IS, insistence on sameness; RSM, repetitive

sensorimotor movements; SI, self-injurious behaviour; CB, compulsive behaviour.

References

Feng, D., Svetnik, V., Coimbra, A., & Baumgartner, R. (2014). A comparison of confidence interval methods for the concordance correlation coefficient and intraclass correlation coefficient with small number of raters. *Journal of biopharmaceutical statistics, 24*(2), from https://pubmed.ncbi.nlm.nih.gov/24605969/.

Grzadzinski, R., Carr, T., Colombi, C., McGuire, K., Dufek, S., Pickles, A., & Lord, C. (2016). Measuring changes in social communication behaviors: Preliminary development of the Brief Observation of Social Communication Change (BOSCC). *Journal of Autism and Developmental Disorders, 46*(7), 2464–2479.

Jacobson, N., & Truax, P. (1991). Clinical significance: A statistical approach to denning meaningful change in psychotherapy research. *Journal of Consulting and Clinical Psychology, 59*(1), 12–19.

Janvier, D., Choi, Y. B., Klein, C., Lord, C., & Kim, S. H. (2022). Brief Report: Examining Test-Retest Reliability of the Autism Diagnostic Observation Schedule (ADOS-2) Calibrated Severity Scores (CSS). *Journal of Autism and Developmental Disorders, 52*(3), 1388–1394.

Kästel, I. S., Vllasaliu, L., Wellnitz, S., Cholemkery, H., Freitag, C. M., & Bast, N. (2021). Repetitive behavior in children and adolescents: Psychometric properties of the German version of the Repetitive Behavior Scale-Revised. *Journal of Autism and Developmental Disorders, 51*(4), 1224–1237.

Shrout, P. E., & Fleiss, J. L. (1979). Intraclass correlations: uses in assessing rater reliability. *Psychological bulletin, 86*(2), from https://pubmed.ncbi.nlm.nih.gov/18839484/.
